# Metagenomic analysis of gut microbiome and resistome of Whooper and Black Swans: a one health perspective

**DOI:** 10.1186/s12864-023-09742-2

**Published:** 2023-10-24

**Authors:** Yin Fu, Kaihui Zhang, Fa Shan, Junqiang Li, Yilin Wang, Xiaoying Li, Huiyan Xu, Ziyang Qin, Longxian Zhang

**Affiliations:** 1 College of Veterinary Medicine, Henan Agricultural University, No. 15 Longzihu University Area, Zhengzhou New District, Zhengzhou, 450046 China; 2 International Joint Research Laboratory for Zoonotic Diseases of Henan, Zhengzhou, 450046 China; 3 Ministry of Agriculture and Rural Areas Key Laboratory for Quality and Safety Control of Poultry Products, Zhengzhou, 450046 China; 4 College of Veterinary Medicine, Northwest A&F University, Yangling, 712100 China

**Keywords:** Whooper swan, Black swan, Microbiome, Resistome

## Abstract

**Background:**

With the promotion of “One Health,” the health of animals and their impact on the environment have become major concerns recently. Widely distributed in China, the whooper swans (*Cygnus cygnus*) and black swans (*Cygnus atratus*) are not only important to the ecological environment, but they may also potentially influence public health security. The metagenomic approach was adopted to uncover the impacts of the gut microbiota of swans on host and public health.

**Results:**

In this study, the intestinal microbiome and resistome of migratory whooper swans and captive-bred black swans were identified. The results revealed similar gut microbes and functional compositions in whooper and black swans. Interestingly, different bacteria and probiotics were enriched by overwintering whooper swans. We also found that *Acinetobacter* and *Escherichia* were significantly enriched in early wintering period swans and that clinically important pathogens were more abundant in black swans. Whooper swans and black swans are potential reservoirs of antibiotic resistance genes (ARGs) and novel ARGs, and the abundance of novel ARGs in whooper swans was significantly higher than that in black swans. Metagenomic assembly–based host tracking revealed that most ARG-carrying contigs originated from *Proteobacteria* (mainly *Gammaproteobacteria*).

**Conclusions:**

The results revealed spatiotemporal changes in microbiome and resistome in swans, providing a reference for safeguarding public health security and preventing animal epidemics.

**Supplementary Information:**

The online version contains supplementary material available at 10.1186/s12864-023-09742-2.

## Introduction

With the popularity of the concept of “One Health,” the impact of animals on human and public health security has received increasing attention in recent years [1]. The gut microbiota is important for host health and public health security. They play important roles in food digestion, energy metabolism, immune homeostasis, bacterial enteric infection, and other physiological activities [2–6]. Birds are probably the most abundant and competent vertebrate vectors, as migratory species can mediate the long-distance dispersal or international transfer of antibiotic-resistant bacteria [7]. Various pathogens and antibiotic-resistant genes (ARGs) in the intestinal tracts of birds may spread to the environment through stools [8–10]. Studies have found that migratory birds are a major source of antibiotic-resistant bacteria in the environment [11, 12]. The migrating sandhill crane (*Grus canadensis*) was reported to be associated with outbreaks of *Campylobacter jejuni* [13]. The importance of birds for public health and safety needs attention.

Whooper swans and black swans inhabiting water sources and lakes are important from ecological and economic perspectives. Whooper swan is one of the main migratory birds passing through various parts of China [14]. The black swan is a natural species in Australia that is imported to China as a popular ornamental animal [15]. Projects and studies have focused on describing the microbiota of humans and other mammalian animals, but there are limited studies on the gut microbiome and resistome of swans [14, 16, 17]. Whooper swans are considered reservoirs of ARGs, whereas black swans have been poorly reported, and very little is known about the impact of whooper and black swans on the entire environment [12, 18, 19].

As whooper and black swans live in different regions of China, the microbiome and resistome may change depending on the living conditions. In this study, whole-metagenome shotgun sequencing was used to characterize the microbiome and resistome of whooper and black swans. Furthermore, changes in the microbiome and resistome of migratory whooper swans were uncovered.

## Sample collection

Whooper swans migrate to Sanmenxia in November each year and move away in March the following year. In this study, 21 fecal samples were collected from two groups of overwintering whooper swans. Samples were collected from whooper swans during the early wintering period (December 2019), middle wintering period (January 2020 and January 2021), and late wintering period (February 2021). Seven fresh fecal samples from ornamental black swans were collected from two artificial lakes in Zhengzhou, China (Fig. S5). Fecal samples weighing approximately 1 g were collected from fecal balls near whooper and black swans. Care was taken to avoid fecal material touching the ground [17]. The collected samples were transported to the laboratory using dry ice, soaked in liquid nitrogen for 30 min, and stored at − 80 °C until further analysis.

### DNA extraction and sequencing

DNA extraction and sequencing were performed as previously described [48]. Briefly, fecal metagenomic DNA was extracted using a QIAamp Fast DNA Stool Mini Kit (QIAGEN, Germany). The 1% agarose gel was used to analyze the degree of degradation and potential contamination of metagenomic DNA. DNA purity and concentration were measured using a NanoPhotometer® spectrophotometer (IMPLEN, CA, USA) and the Qubit® dsDNA Assay Kit in a Qubit® 2.0 Fluorometer (Life Technologies, CA, USA), respectively. Metagenomic DNA was sequenced using an Illumina PE150 platform (Novogene, Tianjin, China) (Table S2).

### DNA sequence assembly and annotation

Raw data from the Illumina PE150 sequencing platform were pre-processed using Readfq (V8, https://github.com/cjfields/readfq). The host sequence was removed from raw data using Bowtie2 (V2.4.5) [49] and assembled using MEGAHIT (V1.2.9) [50] to obtain the scaftigs. Scaftigs (≥ 500 bp) were used to predict the open reading frame (ORF) using MetaGeneMark (V3.38) [51] and CD-HIT software (V4.5.8) [52]. To determine the relative abundance of each gene, high-quality reads from the sample were aligned against the gene catalog using SoapAligner (V2.21) [53]. The corresponding relative abundance of each gene (*Ai*) was calculated using the formula: *Ai* = *Ci*/${\sum }_{i=1}^{n}Ci$ (where *Ni* represents the number of reads mapped to each gene and *Li* represents the length of each gene; *Ci* = *Ni*/*Li*) [12]. The obtained unigenes were used to BLAST the sequences from the NCBI NR database (V202012, https://www.ncbi.nlm.nih.gov/) using DIAMOND software (V2.0.14) [54]. We used the lowest common ancestor (LCA) algorithm to obtain the number of genes and abundance information for each sample in each taxonomic hierarchy (kingdom, phylum, class, order, family, genus, and species) [55]. DIAMOND software was used to blast unigenes to functional databases, including eggNOG (V5.0, http://eggnogdb.embl.de/) and KEGG (V202201, http://www.kegg.jp/kegg/) [56], and the best BLAST hit was used for subsequent analysis. The unigenes were blasted against the CARD database using DIAMOND software to analyze the resistance genes [57]. To ensure the accuracy of ARGs, an 80% identity cutoff was selected as the search criterion [12].

### Identification of iMGE

All ORFs with resistance genes were annotated using the NR database. As described previously, the ORFs co-located with resistance genes were identified as iMGE by string-matching their annotations using the following keywords: “transposase,” “transposon,” “conjugative,” “integrase,” “integron,” “recombinase,” “resolvase,” “conjugal,” “mobilization,” “recombination,” and “plasmid” [58, 59].

### Statistical analysis and visualization

Venn diagrams, alpha diversity (Chao1 and Shannon indices) and principal co-ordinates analysis (PCoA) based on Bray–Curtis were calculated and plotted by the Tutools platform (https://www.cloudtutu.com); LEfSe score was analyzed by galaxy platform (http://huttenhower.sph.harvard.edu/galaxy/) [60]; The distribution of ARGs in bacteria of different taxonomic levels was plotted as a Sankey diagram using the networkD3 package (https://cran.r-project.org/web/packages/networkD3) in R (v4.2.0). Phylogenetic trees, stack bar diagrams, and pie charts were constructed on a chiplot platform (https://www.chiplot.online/).

## Results

### Similar gut microbiome and function genes exist in whooper swan and black swan

**Fig. 1:**
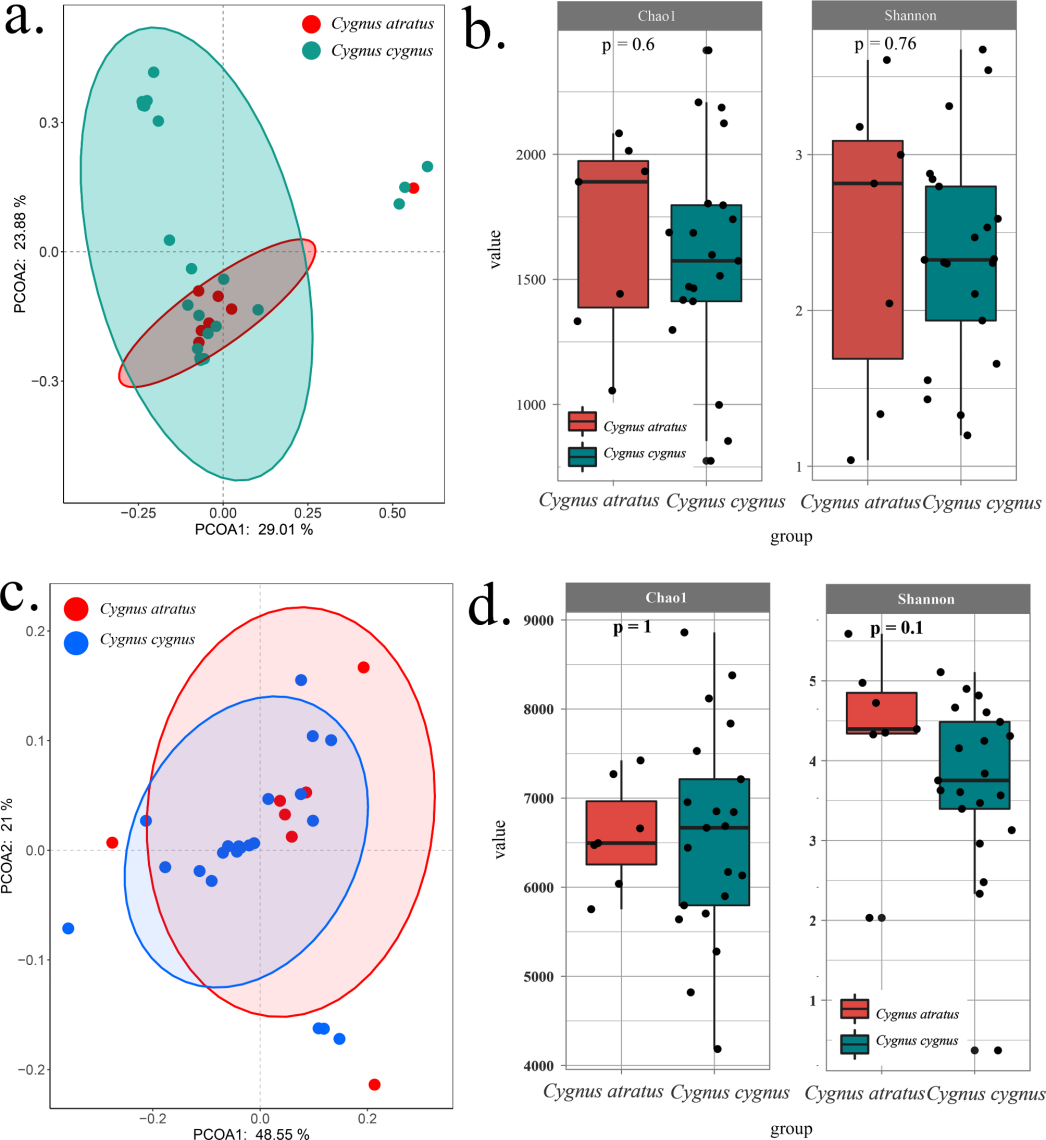
Comparison of gut microbiota and functional genes between whooper swans and black swans. **(a)** PCoA of gut microbiota. **(b)** Alpha diversity (Chao1 and Shannon indices) of gut microbiota. **(c)** PCoA of the functional genes. **(d)** alpha diversity (Chao1 and Shannon index) of functional genes. The PCoA was based on the Bray–Curtis distance. Boxes of alpha diversity denote the interquartile range (IQR) between the first and third quartiles (25th and 75th percentiles, respectively), and the line inside denotes the median. Whiskers denote the lowest and highest values within 1.5 times and the IQR from the first and third quartiles, respectively

At the phylum level, *Firmicutes*, *Proteobacteria*, and *Bacteroidetes* were the dominant phyla in both whooper swans and black swans (Fig. S1). Alpha diversity analyses showed that the richness and diversity of the gut microbiota were similar between whooper swans and black swans (Fig. 1b). Moreover, beta diversity analyses of gut microbiota showed that most whooper swans and black swans had similar gut microbial structures, but many whooper swans clustered separately (Fig. 1a). Consequently, their functional genes were found to be similar and showed a lower discrepancy between individuals (Fig. 1c and d). The NR genes of the gut microbiota were annotated based on the eggNOG and KEGG databases, and the functional pathways of replication, recombination, and repair (9.2%), amino acid transport and metabolism (5.1%), and carbohydrate transport and metabolism (4.7%) were relatively more abundant among the known functions (Fig. S2).

### Variations of gut microbiota in overwintering whooper swans

**Fig. 2:**
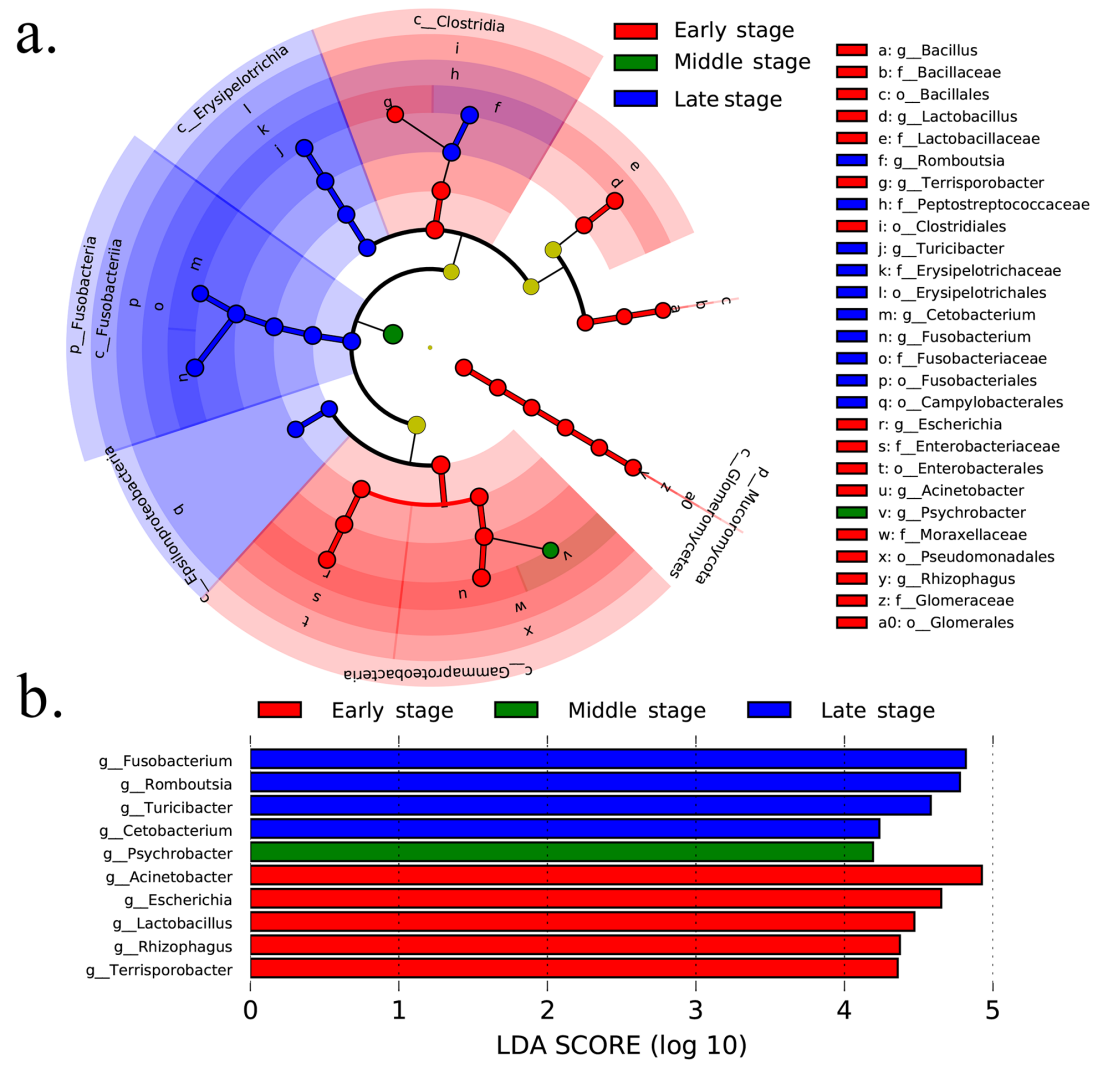
Biomarkers of intestinal microbes in whooper swans during overwintering. **(a)** Cladogram diagram showing the gut microbiota with significant differences among the three groups. Red, green, and blue indicate different groups, with the species classification at the phylum, class, order, family, and genus levels shown from inside to outside. **(b)** Plot of LEfSe data: The length of the bar column represents the LDA score

The taxa that most likely explained the differences between whooper swans from different wintering times were defined by linear discriminant analysis effect size (LEfSe). The results showed cladograms representing the potential biomarkers of different groups. Most biomarkers were significantly enriched in the early and late wintering periods (Fig. 2a). At the genus level, *Terrisporobacter*, *Rhizophagus*, *Lactobacillus*, *Escherichia*, and *Acinetobacter* showed significant enrichment during early wintering periods; *Psychrobacter* showed significant enrichment during middle wintering periods; and *Cetobacterium*, *Turicibacter*, *Romboutsia*, and *Fusobacterium* showed significant enrichment during the late wintering periods (linear discriminant analysis (LDA) score > 4.0, P < 0.05) (Fig. 2b).

### Whooper swans and black swans are potential carriers of pathogens

**Fig. 3:**
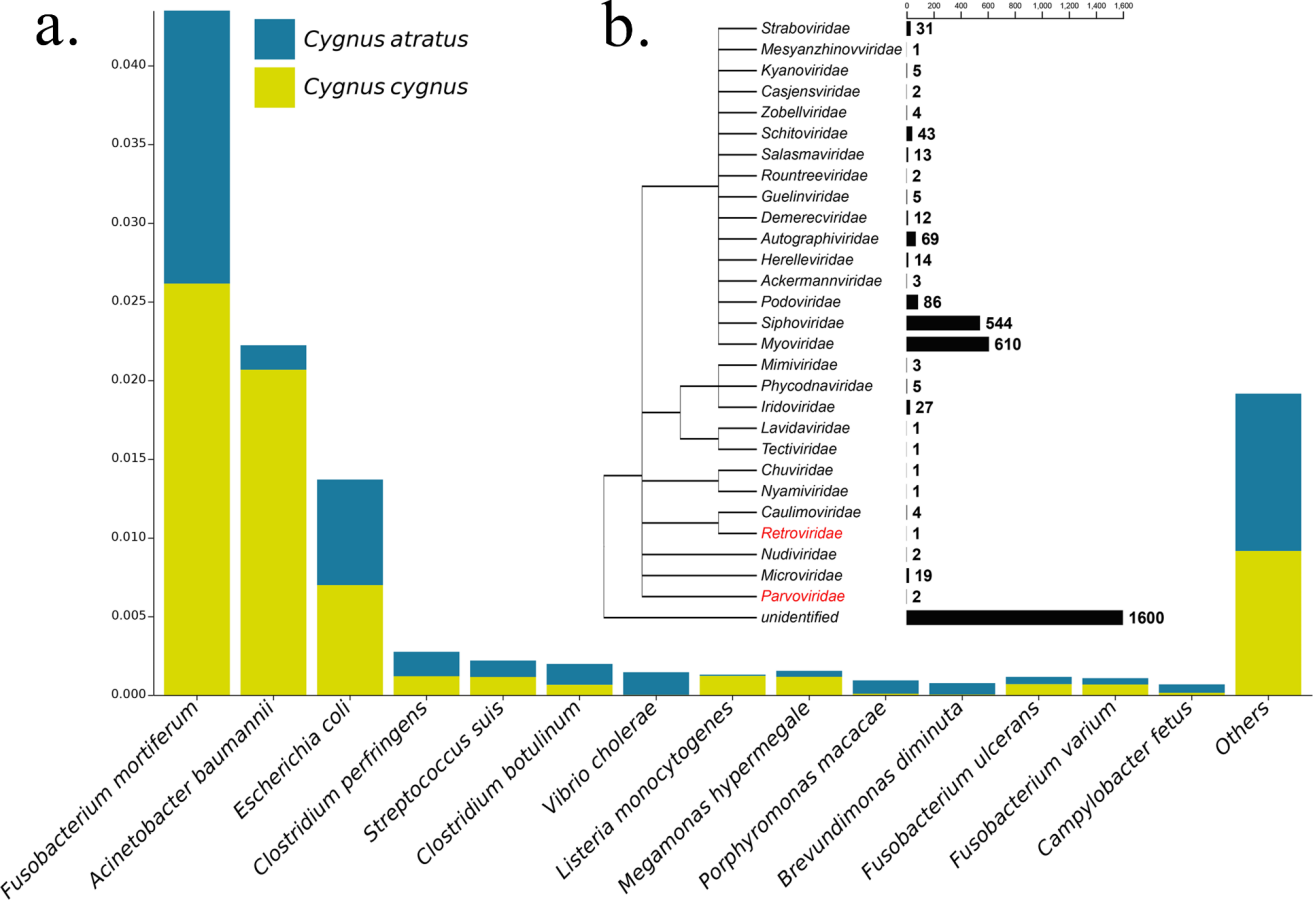
Distribution of pathogens in whooper and black swans. **(a)** Relative abundance of the top 10 germs in whooper swans and black swans. **(b)** Overall abundance of viral families identified in whooper and black swans. Families of viruses that can cause disease in animals are marked in red. The results were analyzed and visualized using the taxonomy database of the National Center for Biotechnology Information (NCBI). The length of the bar corresponds to the total number of reads in 28 samples

Zoonotic germs were detected using a previously published pathogen list [20]. The average relative abundance of most opportunistic pathogens was less than 1%. Among the top 10 most abundant opportunistic genera, *Fusobacterium mortiferum*, *Acinetobacter baumannii*, and *Escherichia coli* were the most prevalent pathogens. Overall, the abundance of pathogens in whooper swans was higher than that in black swans; however, some clinically important pathogens, such as *Clostridium perfringens*, *Clostridium botulinum*, *Vibrio cholerae*, and *Campylobacter fetus*, were more abundant in black swans (Fig. 3a).

A total of 3111 NR genes were annotated for 28 different virus families; 47.1% of NR genes were belong to bacterial viruses (Fig. 3b), and two bird viruses (*Avian leukosis virus* and *Pigeon parvovirus*) were recorded in black swans. Enteric protozoan parasites such as *Eimeria* and *Isospora* were also identified. *Isospora manorinae* was exclusively found in whooper swans, whereas *Isospora superbusi* and *Eimeria vejdovskyi* were found only in black swans. *Isospora amphiboluri* and *Eimeria brunetti* were detected in both whooper and black swans (Table 1).

**Table 1:** Virus and parasites detection rates in whooper swan and black swan

Organism	Spices	Hosts	
		Whooper swan	Black swan
Virus	*Avian leukosis virus*	0/21	1/7
	*Pigeon parvovirus*	0/21	1/7
Parasites	*Eimeria brunetti*	2/21	3/7
	*Eimeria vejdovskyi*	0/21	2/7
	*Isospora amphiboluri*	1/21	3/7
	*Isospora superbusi*	0/21	3/7
	*Isospora manorinae*	2/21	0/7

**Table 2:** Detection rate of ARGs with iMGEs in whooper swan and black swan

ARGs	iMGEs	Hosts	
		Whooper swan	Black swan
*tetD*	transposon and transposase	6/21	3/7
*CARB-1*	transposon	4/21	3/7
*H-NS*	integrase	11/21	4/7
*tetW*	transposon	20/21	7/7

### Whooper swans and black swans as potential reservoirs of ARGs

A total of 297 NR genes greater than or equal to 85% of the target sequence length were identified in the CARD database [12] and recognized as antimicrobial resistance protein-coding genes. Of the 297 antimicrobial resistance protein-coding genes, 195 genes had over 80.0% amino acid identity, and the other 102 genes were considered novel antimicrobial resistance genes [12], with an amino acid identity ranging from 40.7 to 79.9%. It is worth mentioning that the *mcr-1* gene found in a black swan had 100% nucleotide identity (Table S1).

The 297 NR genes were grouped into 164 ARGs. All ARGs were matched to 45 corresponding antibiotics, which conferred resistance to almost all the major antibiotic classes commonly administered for clinical and agricultural use. The ARGs were mainly related to aminoglycoside, tetracycline, and multidrug resistance and correspondingly had a higher relative abundance. However, fluoroquinolone-tetracycline has fewer ARG types but a higher relative abundance (Fig. S3).

### Carrier of ARGs and transfer risk

**Fig. 4:**
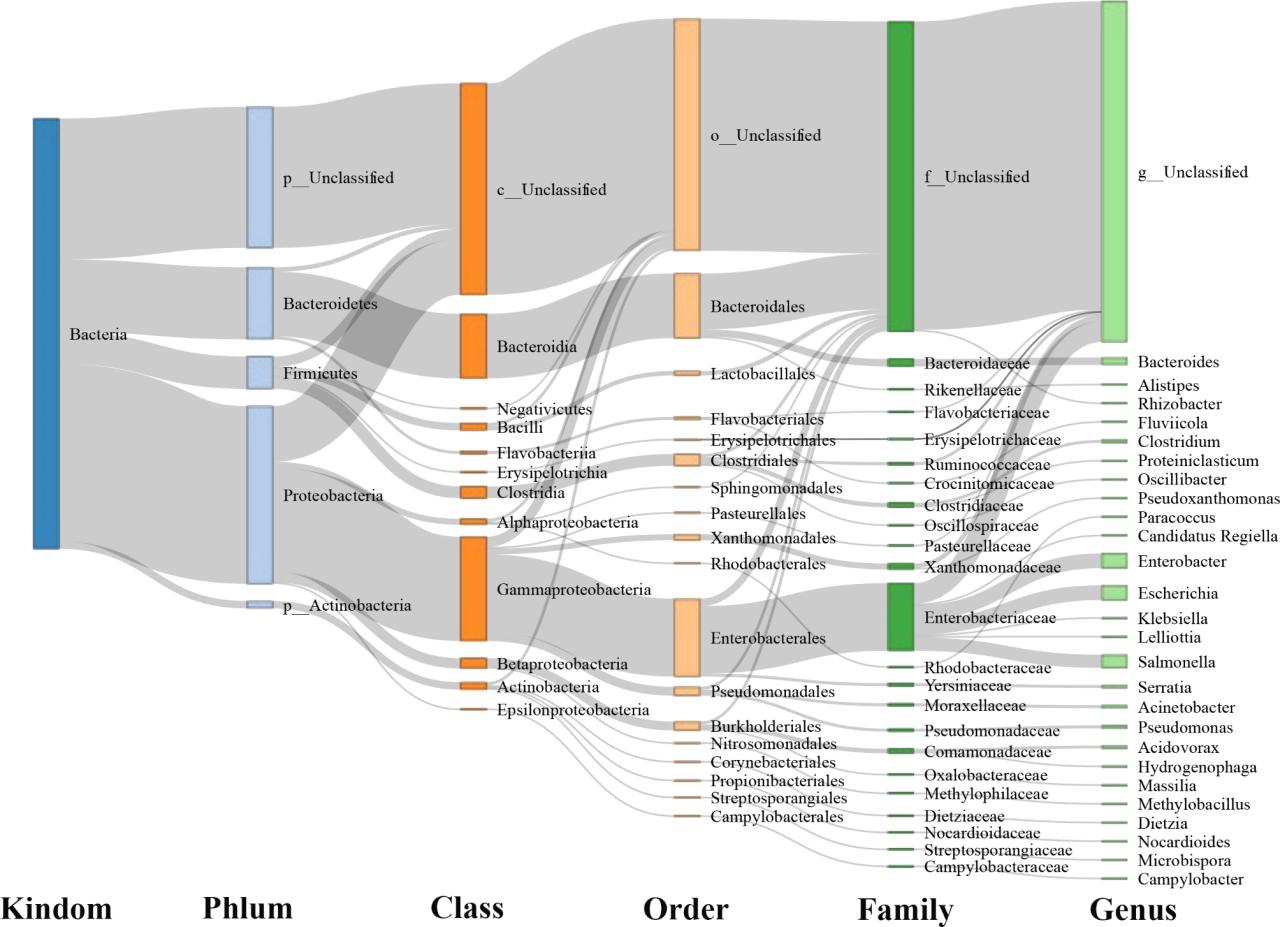
Distribution of antimicrobial resistance protein-coding genes in bacteria at different taxonomic levels. The rectangles represent different taxonomic levels. The height of the rectangles indicates the number of ARGs

The 297 NR genes annotated as ARGs were aligned to the NCBI NR database to trace the bacteria and determine whether they possibly integrated ARGs. The results revealed that the ARGs were from *Proteobacteria* (mainly *Gammaproteobacteria*), *Bacteroidetes* (mainly *Bacteroidia*), and *Firmicutes* (mainly *Bacilli* and *Clostridia*) (Fig. 4). Four NR genes of ARGs associated with mobile genetic elements (iMGEs) had a higher prevalence. The iMGEs associated with ARGs in the gut microbiome of whooper and black swans may promote the dissemination of resistance via horizontal gene transfer (HGT) among a diverse range of hosts (Table 2).

### Distribution characteristics of ARGs in whooper swan and black swan

**Fig. 5:**
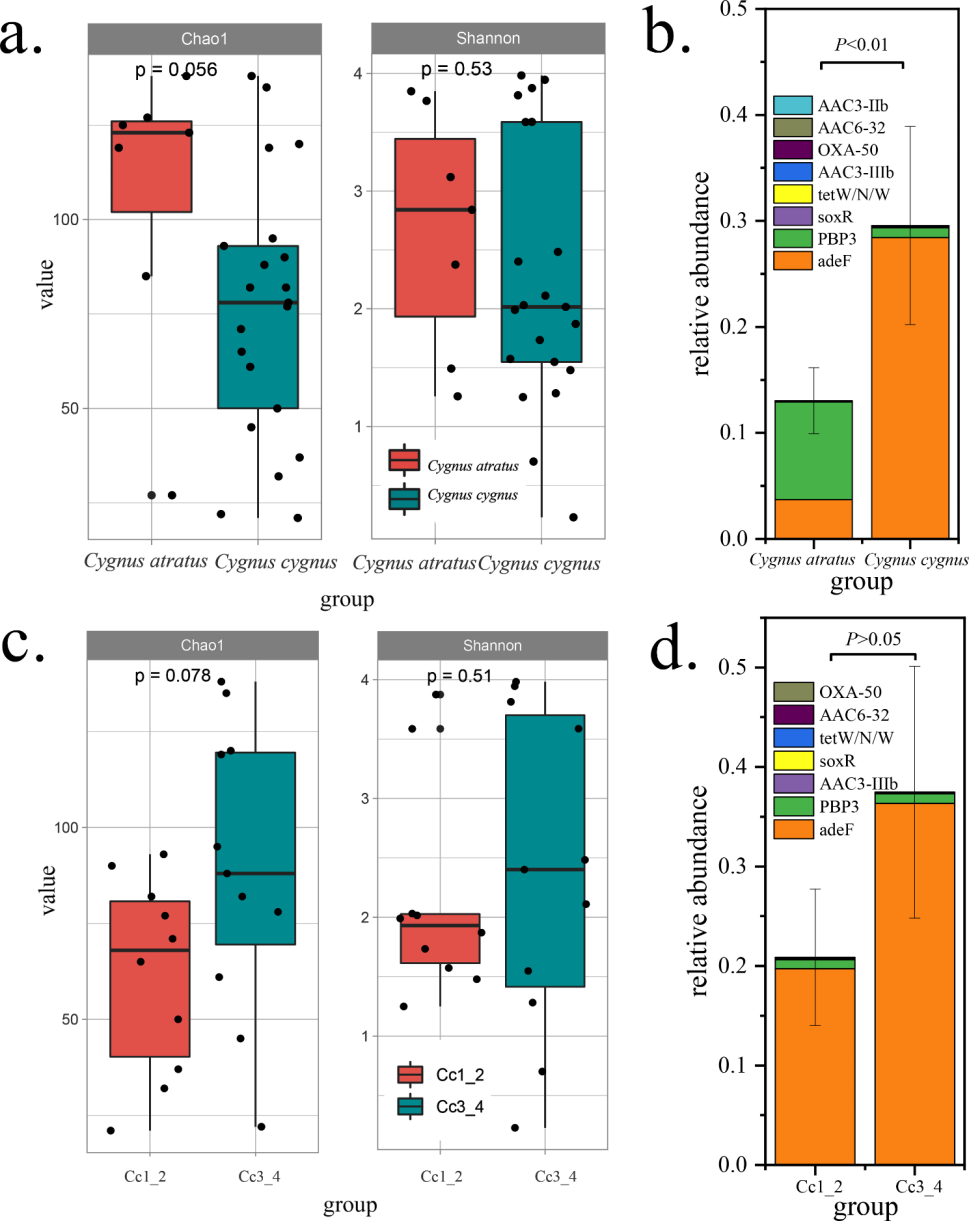
Different ARGs and novel ARGs between whooper swans and black swans and between whooper swans from different years. **(a)** Alpha diversity (Chao1 and Shannon index) of ARGs between whooper swans and black swans. **(b)** Relative abundance difference of novel ARGs between whooper swans and black swans. **(c)** Alpha diversity (Chao1 and Shannon index) of ARGs between whooper swans from different years. **(d)** Relative abundance of novel ARGs genes in whooper swans from different years. Boxes of alpha diversity denote the interquartile range (IQR) between the first and third quartiles (25th and 75th percentiles, respectively), and the line inside denotes the median. Whiskers denote the lowest and highest values within 1.5 times and the IQR from the first and third quartiles, respectively. The whiskers in the histogram denote the standard error, and the *P*-value was analyzed using a homogeneity test of variance

Whooper swans had a higher diversity and richness of ARGs than black swans, but the difference was not statistically significant (Fig. 5a). However, the relative abundance of novel ARGs was significantly higher in whooper swans than in black swans, and the novel *adeF* genes were more enriched in whooper swans, while the novel *PBP3* genes were more enriched in black swans (Fig. 5b).

The diversity and richness of ARGs in the two groups of whooper swans were similar (Fig. 5c), and the relative abundance of novel ARGs was not significantly different (Fig. 5d). However, the biomarker demonstrated that all 25 ARGs were significantly enriched in the samples from the second year (LDA > 2, P < 0.05), and that the novel ARGs had a higher relative abundance in the samples from the second year (Fig. S4). With the migration of whooper swans, the composition of intestinal resistome changes, and concomitantly, some ARGs accumulate, mainly aminoglycoside antibiotic and tetracycline antibiotic genes.

## Discussion

Whooper and black swans are large waterfowl of the genus *Cygnus*, widely distributed in various parts of China. They share areas associated with human activities and have important ecological and economic significance. *Firmicutes*, *Proteobacteria*, and *Bacteroidetes*, account for the largest proportion of the gut microbiota among whooper and black swans, which is similar to previous studies in swans [12, 14, 21]. Many waterbirds have different characteristics in relation to the intestinal microbiota, such as *Bacteroidetes*, which has a higher proportion in *Alba alba* and *Tringa nebularia*, but more abundant *Actinobacteria* in *Anser indicus* [12]. The repertoire of the intestinal microflora of birds is directly related to their species, dietary composition, and living environment [22–24]. Whooper and black swans belong to the same genus and share similar habits; therefore, it is unsurprising that they harbor similar intestinal microbes.

The gut microbiota plays vital roles in host metabolism, nutrition, physiology, immune function, and disease resistance [2–6, 16]. In this study, *Acinetobacter* and *Escherichia* were found to be significantly enriched in early wintering period swans, which is associated with various diseases [25–27]. Along with migration, changes in lifestyle and dietary selective pressure may destroy stable intestinal microbiota, leading to physiological stress, which further results in a decline in immune function, thereby increasing the abundance of pathogenic bacteria [14]. *Lactobacillus*, *Terrisporobacter*, and *Clostridium* are significantly enriched in swans in the early wintering period and facilitate health maintenance and survival in harsh environments [28–30]. Significantly enriched *Rhizophagus* in swans during early wintering periods indicates that roots are their main food source [31]. Similarly, the presence of enriched *Turicibacter* and *Romboutsia*, short-chain fatty acid-producing bacteria, in the late wintering period group shows their association with energy expenditure and contribution to intramuscular adipogenesis [32, 33]. *Turicibacter* and *Romboutsia* may be related to energy accumulation before the migration of whooper swans during late overwintering. Some intestinal probiotics may create a huge promotion to help the whooper swan adapt to the environment and complete migration.

Abundant germs were found in whooper swans and black swans, and some clinically important pathogens, such as *Clostridium perfringens*, *Clostridium botulinum*, *Vibrio cholerae*, and *Campylobacter fetus*, were more abundant. Birds carry a variety of opportunistic pathogens; therefore, some foodborne disease outbreaks are thought to be associated with birds [13, 34]. However, a comprehensive understanding of the involvement of wild birds in transmitting enteric bacteria to humans is lacking [35]. Opportunistic pathogens are widely distributed in the environment and gut of animals and can most often be symbiotic with animals [20, 36]. The role of birds, particularly migratory birds, in the spread of diseases is of great concern. Viruses and parasites found in this study could harm animals and cause huge losses to the poultry industry [37, 38]. Previous studies have shown that the highly pathogenic avian influenza H5N1 virus breaks out among migratory birds and may spread along migratory bird routes [19]. Whooper and black swans are potential reservoirs and sources of pathogens.

The emergence of multiple antibiotic-resistant bacteria and widespread ARGs has led to the emergence of environmental pollutants [39]. The most prevalent classes of ARGs in whooper swans and black swans are tetracycline, lincosamide, and aminoglycoside, which are common in humans and domestic animals [40]. Bacteria have a rich natural history of resistance [41], and it is difficult to find birds that have never been exposed to antibiotic-polluted environments. The variety and abundance of resistance genes in whooper swans increased compared to those in the previous year. Numerous studies have revealed that ARGs can spread from the environment to birds [42, 43], and the increase in ARGs in whooper swans may be related to environmental sources during migration. In particular, ARGs in feces may enter the environment, and studies have shown that migratory birds are a major source of environmental antibiotic resistance in their habitats [11]. Migrating birds may even distribute antibiotic-resistant bacteria and antibiotic-resistant genes to the natural geographically isolated regions far from anthropogenic activities [44].

Colistin is considered the last-resort drug for treating deadly infections caused by multi-resistant Gram-negative bacteria; however, movable colistin resistance (*mcr*) genes jeopardize the efficacy of colistin [45]. It is evident that the *mcr-1* gene is transmitted by plasmids and is widespread in migratory birds, and it was detected in whooper swans in Sanmenxia, China [12]. This study only found *mcr-1* genes in black swans, which may indicate a decreased prevalence of the *mcr* gene in migrating whooper swans [12]. The low detection rate of the *mcr* gene may be related to the scientific use of polymyxin in recent years [46, 47], and it may also be attributed to the small number of samples examined relative to the total number of migrating whooper swans.

## Conclusions

This study used shotgun metagenomic sequencing to compare the gut microbiome and resistome of whooper swans and black swans. We screened potential biomarkers among whooper and black swans at various wintering stages and comprehensively assessed the potential threats to public health security. Moreover, migratory birds may eventually become a greater threat to the environment. Therefore, continuous monitoring of migratory birds in relation to the distribution of resistant pathogens and their genes is necessary to provide timely information regarding the transmission direction of such biological pollutants.

### Electronic supplementary material

Below is the link to the electronic supplementary material.

Supplementary Material 1

Supplementary Material 2

Supplementary Material 3

## Data Availability

All data generated during this study are available at the Sequence Read Archive (SRA) under BioProject number PRJNA890321 (SRR21898906-SRR21898933).

## Abbreviations

ARG: Antibiotic Resistance Gene
HGT: Horizontal Gene Transfer
CARD: Comprehensive Antibiotic Resistance Database
NCBI: National Center for Biotechnology Information
KEGG: Kyoto Encyclopedia of Genes and Genomes
iMGE: Identified as Mobility Indicators
eggNOG: Evolutionary genealogy of genes:Non-supervised Orthologous Groups
PCoA: Principal Co-ordinates Analysis
LEfSe: Linear discriminant analysis Effect Size
IQR: Interquartile
LDA: Linear Discriminant Analysis
LCA: Lowest Common Ancestor
NCBI: National Center for Biotechnology Information
