# Supplementary material for: Metagenomic analysis of gut microbiome and resistome of Whooper and Black Swans: a one health perspective
Source: BMC Genomics. 2023 Oct 24;24:635. doi: 10.1186/s12864-023-09742-2 (PMC10594901; doi:10.1186/s12864-023-09742-2)
Supplement: Supplementary file 3 — Supplementary Material 3 [file 12864_2023_9742_MOESM3_ESM.pdf]

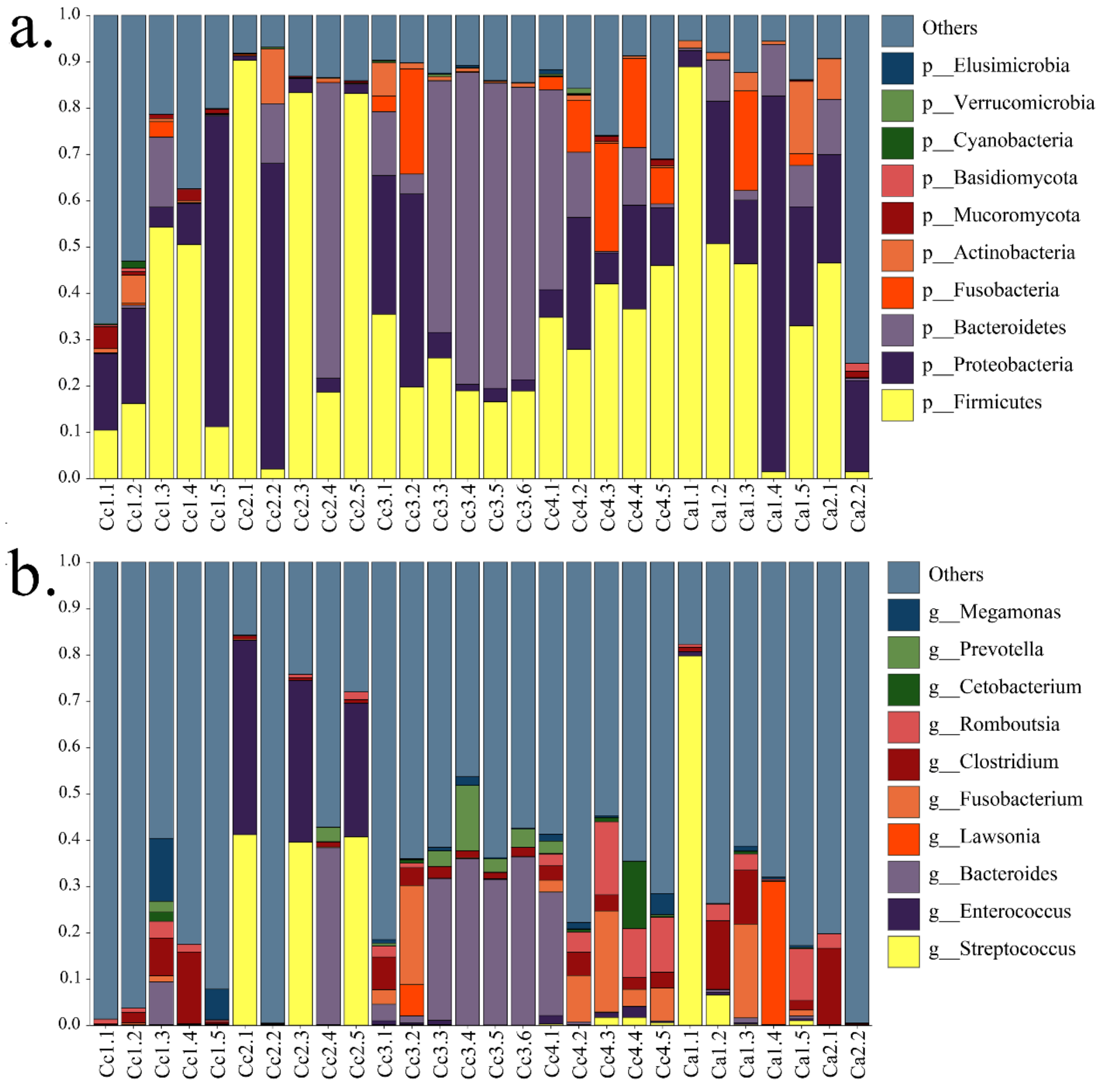

Fig. S1. Relative abundance (%) of the top 10 microbes in samples from whooper swans (Cc1.1-Cc4.5) and black swans (Ca1.1-Ca2.2). **(a)** Phylum levels. **(b)** Genus level.

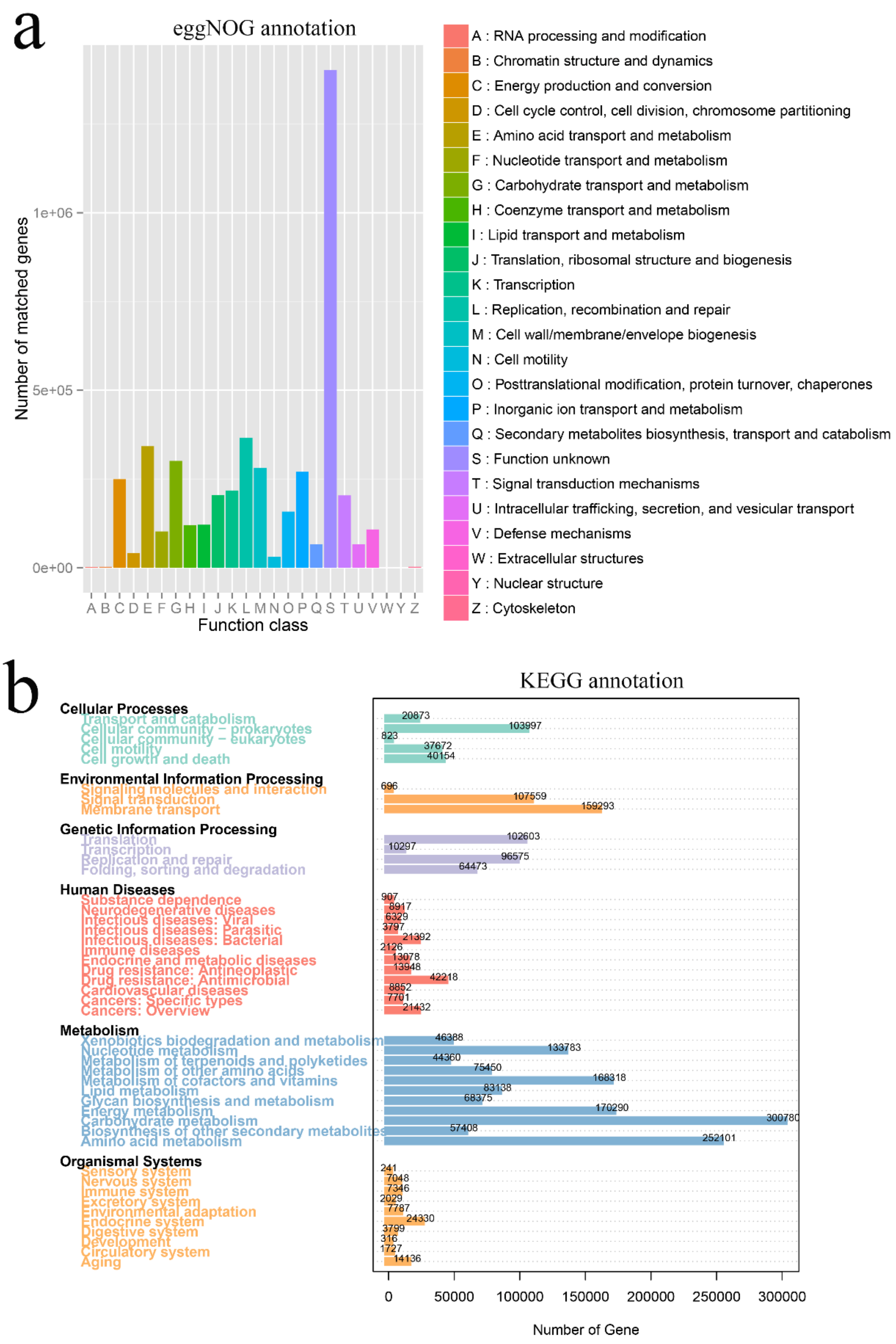

Fig. S2. Functional annotation results. **(a)** Functional annotation of the predicted nonredundant gene catalog based on the eggNOG database. **(b)** Functional annotation of the predicted non-redundant gene catalog based on the KEGG database.

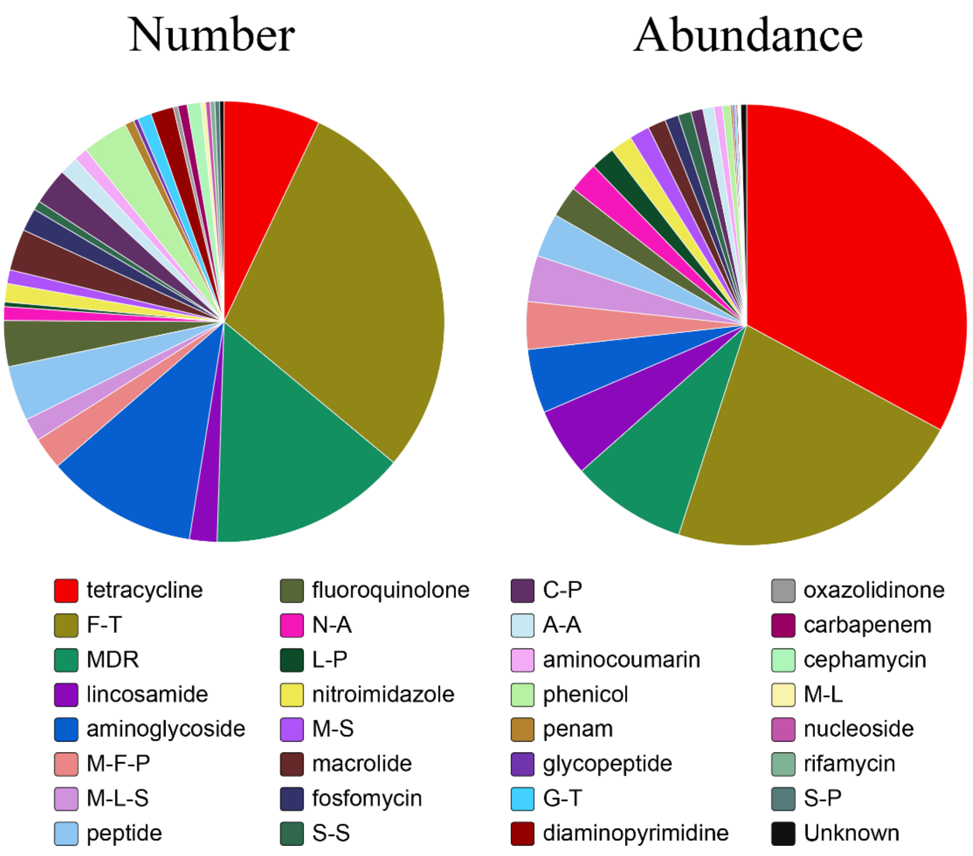

Fig. S3. Abundance and number of ARGs for each antimicrobial. All the ARGs were classified according to the antimicrobials for which they showed resistance. The abbreviated names of the antimicrobial classes are used in the figures, and the corresponding full names are presented in Table S1.

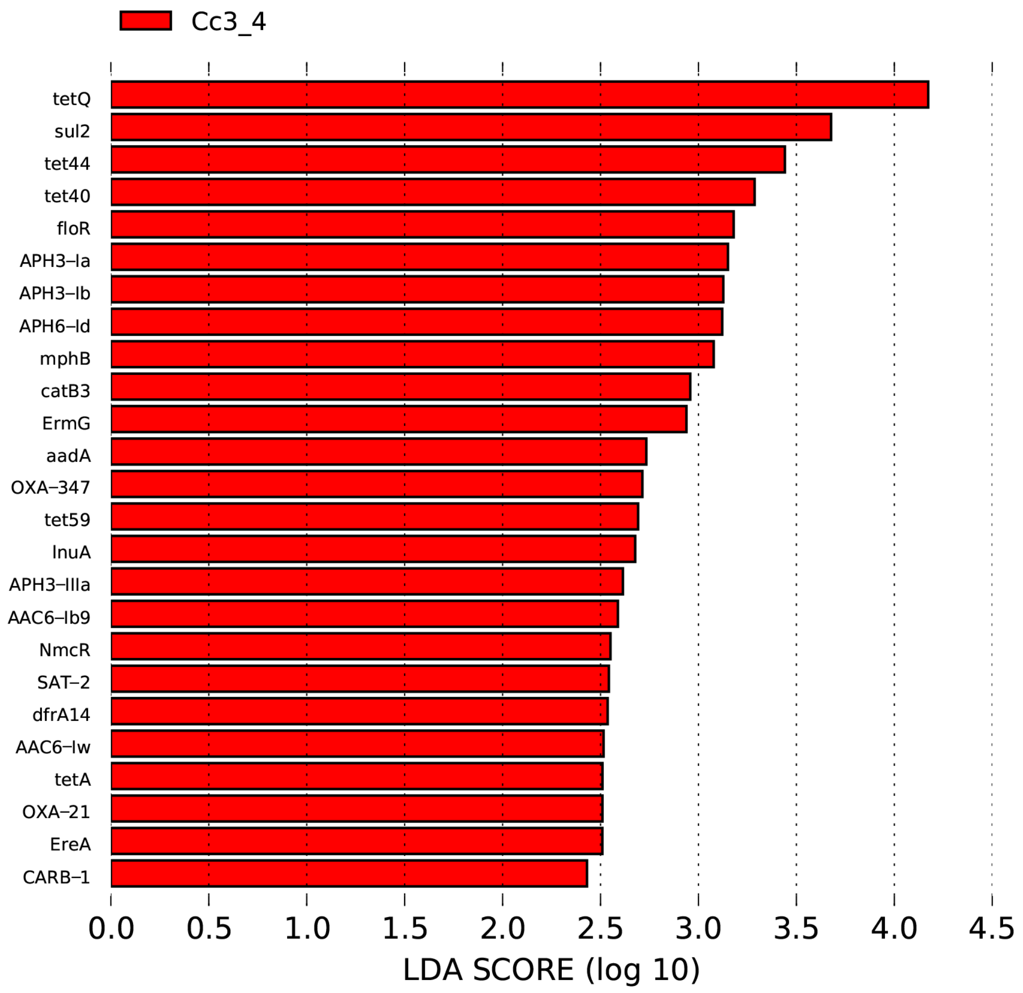

Fig. S4. Plot from LEfSe analysis based on ARGs of whooper swans over two years (Cc1_2 and Cc_3_4); the length of the bar column represents the LDA score.

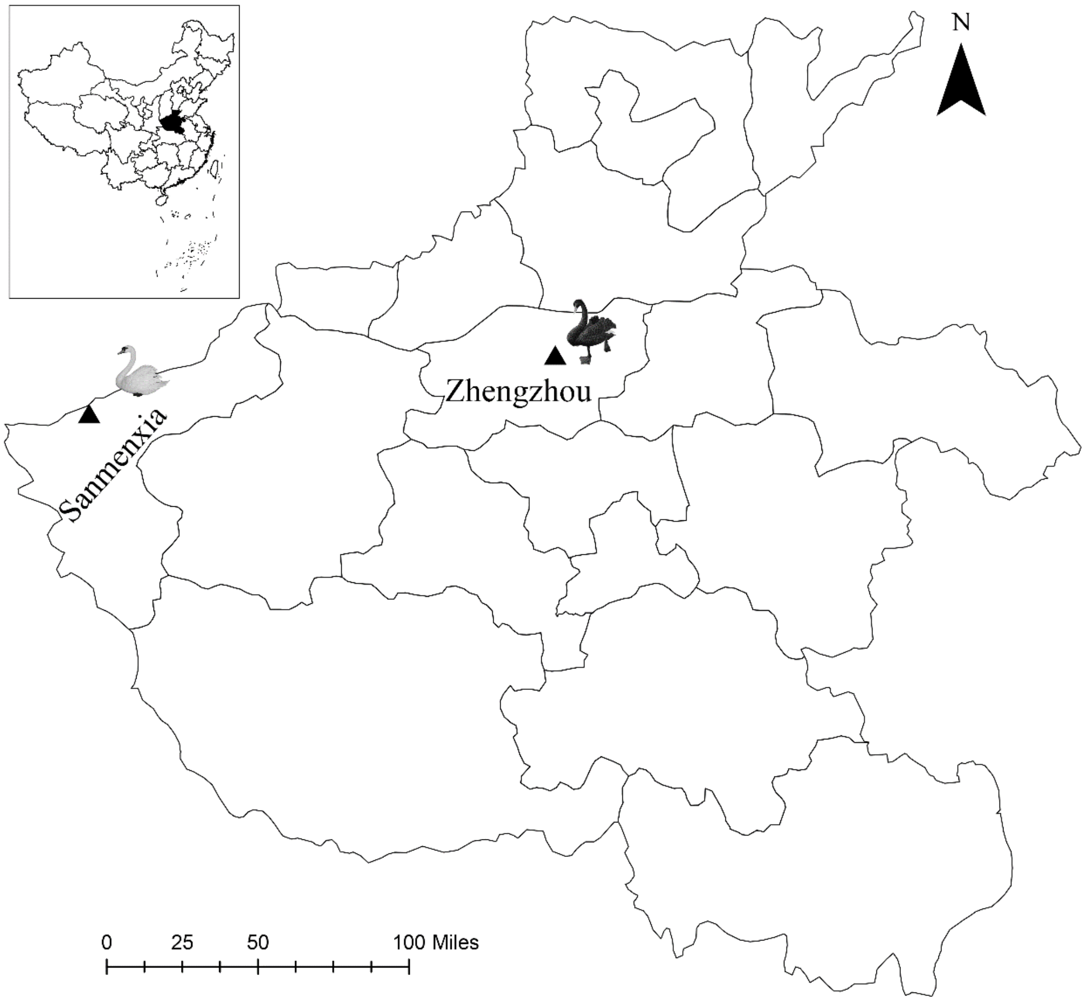

Fig. S5. Geographical distribution and sample information.
